# Expression of E-cadherin, Slug and NCAM and its relationship to tumor invasiveness in patients with acromegaly

**DOI:** 10.1590/1414-431X20176808

**Published:** 2017-12-11

**Authors:** G.A. Mendes, T. Haag, G. Trott, C.G.S.L. Rech, N.P. Ferreira, M.C. Oliveira, M.B. Kohek, J.F.S. Pereira-Lima

**Affiliations:** 1 Programa de Pós-Graduação em Patologia, Universidade Federal de Ciências da Saúde de Porto Alegre, Porto Alegre, RS, Brasil; 2 Centro de Neuroendocrinologia, Santa Casa de Porto Alegre, Universidade Federal de Ciências da Saúde de Porto Alegre, Porto Alegre, RS, Brasil

**Keywords:** Acromegaly, Pituitary neoplasms, Cadherins, Slug, Neural cell adhesion molecules

## Abstract

Pituitary adenomas account for 10–15% of primary intracranial tumors. Growth hormone (GH)-secreting adenomas account for 13% of all pituitary adenomas and cause acromegaly. These tumors can be aggressive, invade surrounding structures and are highly recurrent. The objective of this study was to evaluate E-cadherin, Slug and neural cell adhesion molecule (NCAM) expression in GH-secreting pituitary adenomas and its relationship to tumor invasiveness. A cross–sectional study of patients who underwent hypophysectomy due to GH-secreting pituitary adenoma from April 2007 to December 2014 was carried out. The medical records were reviewed to collect clinical data. Immediately after surgery, tumor samples were frozen in liquid nitrogen and stored in a biofreezer at –80°C for assessment of E-cadherin 1 (*CDH1*), SLUG (*SNAI2*), and NCAM (*NCAM1*) by real-time PCR. The samples were fixed in formalin and embedded in paraffin for immunohistochemical analysis of E-cadherin and NCAM. Thirty-five patients with acromegaly were included in the study. Of these, 65.7% had invasive tumors. Immunohistochemically, E-cadherin was expressed in 96.7% of patients, and NCAM in 80% of patients. There was no statistically significant relationship between tumor grade or invasiveness and immunohistochemical expression of these markers. Regarding gene expression, 50% of cases expressed *CDH1*, none expressed *SNAI2*, and 53.3% expressed *NCAM1*. There was no statistically significant relationship between tumor grade or invasiveness and gene expression of *CDH1*, *SNAI2*, and *NCAM1*. The absence of Slug overexpression and of E-cadherin and NCAM suppression suggests that expression of these markers is not associated with tumor invasiveness in GH-secreting pituitary adenomas.

## Introduction

Pituitary adenomas are common tumors, with an estimated prevalence of 16.7%, and autopsy studies have reported their presence in 20 to 25% of cases, accounting for 10 to 15% of primary intracranial tumors (1). Growth hormone (GH)-secreting pituitary adenomas account for 13% of all pituitary adenomas and lead to acromegaly, a chronic disease characterized by hypersecretion of GH and insulin-like growth factor 1. The mean age at diagnosis of patients with acromegaly is 40 years, with no difference between men and women (2). The disease has cardiovascular, rheumatologic, respiratory and metabolic consequences. The main cause of death is cardiovascular disease, accounting for 60% of cases, while respiratory disease and malignancies account for 25 and 15% of cases, respectively (3).

Pituitary adenomas are considered benign tumors, but they may become aggressive and invade surrounding tissues. Approximately 60% of patients with adenomas greater than 1 cm (macroadenomas) have tumor recurrence after surgical treatment. The mechanisms underlying the pathogenesis of these tumors are unclear and genes classically involved in neoplastic development, such as *TP53* and *KRAS*, rarely show mutations. In addition, the mitosis index is generally low and Ki67 and PCNA, markers of cell proliferation, have little relevance in predicting tumor behavior (4). The absence of a prognostic classification or consensual prognostic markers limits the evaluation of medical strategies for pituitary tumors.

Some studies have shown that loss of adhesion protein expression may be involved in the pathogenesis of pituitary adenomas and contribute to tumor aggressiveness and invasiveness (4–7). Currently, there are few reports in the literature regarding the role of cell adhesion proteins, such as E-cadherin (ECAD), and neural cell adhesion molecule (NCAM) in pituitary adenomas, as well as of SLUG, which is an important transcriptional regulator of ECAD. Studies suggest that loss of ECAD expression is critical for epithelial-mesenchymal transition, a process in which neoplastic cells acquire cellular motility and invasiveness (8). The results are quite controversial regarding the role of these markers in the invasiveness of pituitary adenomas. Some studies indicate an association of markers with tumor invasiveness (6,7,9), while others do not recognize this association (10–12).

The factors involved in the growth and invasiveness of these tumors are not fully understood, but once identified, they will aid in the identification of invasion and recurrence markers and of potential therapeutic targets (13). The aim of the present study was to evaluate E-cadherin, Slug and NCAM expressions in GH-secreting pituitary adenomas and their relationship to the degree of tumor invasiveness.

## Material and Methods

### Patients

This cross-sectional study consisted of 35 consecutive patients with a clinical and laboratory diagnosis of acromegaly who underwent neurosurgery performed by a single surgeon (NPF) at Hospital São José, Complexo Hospitalar Santa Casa de Porto Alegre, Southern Brazil, from April 2007 to December 2014. All patients undergoing the procedure during this period were included in the study. Written informed consent was obtained from all individual participants prior to their inclusion in the study.

The diagnosis of pituitary adenoma was confirmed by anatomopathological examination. The histopathological diagnosis was made by the neuropathologists of the Department of Pathology of the institution in accordance with the World Health Organization (WHO) guidelines. Hematoxylin and eosin (HE)-stained slides were available for all cases to confirm the presence of tumors. The HE-stained slides were used to guide sampling of the tissue of interest. Each adenoma was immunohistochemically stained for six pituitary hormones (GH, PRL, ACTH, FSH, LH, and TSH) using commercially available antibodies. Immunohistochemical analysis was performed using chromogranin A antibody for all cases in order to characterize the pituitary tissue. The patients' medical records were then reviewed to collect data on gender, age, preoperative images and immunohistochemistry. Tumor grade and invasiveness were defined based on magnetic resonance images (MRI 1.5T) or X-ray computed tomography (CT) scans obtained preoperatively and classified according to the criteria proposed by Hardy (13): grade I (microadenomas, <1 cm in diameter), grade II (≥1 cm in diameter, intrasellar or with suprasellar extension without causing bony erosion), grade III (locally invasive tumors that may be associated with diffuse sellar enlargement and bony erosion of the sella turcica), and grade IV (invasive tumors that involve extrasellar structures including bone, hypothalamus, and the cavernous sinus). Grade I and II pituitary adenomas are considered noninvasive tumors, while grade III and IV adenomas are invasive tumors (14).

The study was approved by the Research Ethics Committee of Universidade Federal de Ciências da Saúde de Porto Alegre (UFCSPA), protocol No. 512/230, and conducted in accordance with the provisions of the Declaration of Helsinki.

### Immunohistochemistry

Tumor tissue samples were fixed in 10% buffered formalin for 24 h and embedded in paraffin. Serial 4-µm thick sections were cut for immunohistochemical analysis. To detect protein expression, sections were incubated with the monoclonal anti-ECAD antibody G-10 (sc-8426; Santa Cruz Biotechnology, USA), at a 1:50 dilution, and anti-NCAM antibody [(EPR2566) (ab133345), Abcam, UK], at a 1:300 dilution. The labeled streptavidin-biotin method (LSAB kit + Peroxidase; Dako, USA) was used for detection. Due to technical problems related to the primary antibody, the analysis of SLUG by means of immunohistochemistry was not possible. Endogenous peroxidase activity was blocked using three 10-min baths in 5% hydrogen peroxide (H_2_O_2_) 30 V in methanol. Nonspecific protein binding was blocked using 1% BSA for 30 min. Incubation with primary antibody was performed overnight at 4°C. Incubation with secondary antibody and tertiary antibody was performed for 40 min at room temperature. The primary antibody was replaced with saline to serve as a negative control. Human tonsil was used as a positive control for ECAD and glioma for NCAM. The antigen-antibody binding was visualized with the diaminobenzidine (DAB) chromogen.

A positive expression was defined as plasma membrane staining classified according to a score based on staining intensity and ratio of positive cells after analysis of the slides (7). Staining intensity was scored as 0 (no staining), 1 (weak), 2 (moderate), and 3 (strong). The ratio of positive cells was scored as 0 (0–5% stained cells), 1 (6–10%), 2 (11–50%), 3 (51–80%), and 4 (> 80%). The final score was obtained by multiplying the intensity ratio by the positive cell ratio: 0 (−, negative expression), 1–3 (+, weak expression), 4–6 (++, moderate expression), and > 6 (+++, strong expression). The slides were read by two independent observers by optical microscopy. The observers were blind to the tumor characteristics.

### Quantitative real-time PCR (qRT-PCR)

Tumor fragments were obtained immediately after surgery, frozen in liquid nitrogen, and stored in a biofreezer at -80°C. Total RNA was extracted using TriReagent (Ludwig Biotec, Brazil), according to the manufacturer's instructions. RNA was then reverse transcribed into cDNA at a final volume of 21 µL using the SuperScript III First-Strand Synthesis System (Invitrogen, USA), according to the manufacturer's instructions. The resulting cDNA samples were diluted to a final concentration of 250 ng/µL for qRT-PCR of ECAD (*CDH1*) and SLUG (*SNAI2*), and of 100 ng/µL for qRT-PCR of NCAM (*NCAM1*). The samples were amplified using Sybr Green (Applied Biosystems, USA) in a total reaction volume of 15 µL under the following conditions: initial denaturation at 50°C for 2 min and at 95°C for 10 min followed by 40 cycles at 95°C for 15 s and 60°C for 1 min. The following primers were used: *CDH1* (forward: 5′-GCCGAGAGCTACACGTTCAC-3′, reverse: 5′-ACTTTGAATCGG GTGTCGAG-3′), *SNAI2* (forward: 5′-ATATTCGGACCCACACATTACC-3′, reverse: 5′-ACATTCTGGAGAAGGTTTTGGA-3′), and *NCAM1* (forward: 5′-AACAAAGCATGATGGGTGAA-3′, reverse: 5′-GTCTGTGGTGTTGGAAATGC-3′). All reactions were run in duplicate using a StepOnePlus system (Applied Biosystems, USA). Samples without cDNA were used as negative controls. As an endogenous control, *GAPDH* was used as a reference for normalization (forward: 5′-GGAAGGTGAAGGTCGGAGTCA-3′, reverse: 5′-GTCATTGATGGCAACAATATCCACT-3′). *GAPDH* was amplified by qRT-PCR for each sample and for each RT-negative control, with the same specified conditions for gene analysis. A cycle threshold (Ct) <40 was classified as showing good quality cDNA. Commercially available pooled RNA (Human Pituitary Gland Pool of RNA-636157, Clontech Laboratories, USA), consisting of 39 healthy pituitary glands from adult males and females, was used for calibration of qRT-PCR. Data were converted to normalize expression ratios using the Applied Biosystems-recommended 2^(−ΔΔCt) method. Relative expression (the normalized target concentration related to the endogenous reference) was given by the 2^(−ΔΔCt) formula, where: ΔΔCt = [(Ct target gene – Ct *GAPDH* gene in samples) – (Ct normal pituitary target gene – Ct *GAPDH* normal pituitary gland pool)]. Data from tumor tissues are reported as equal to 1 (reference level). An expression level < 1 was defined as lower expression compared to normal pituitary gland pool and ≥1 as presence of equal or higher expression compared to normal pituitary gland pool (15).

### Statistical analysis

Descriptive statistical analysis and normality testing of the data (Shapiro-Wilk test) were performed to determine the distribution of the data. Quantitative variables are reported as means±SD or medians according to their distribution. Age was symmetrically distributed and reported as mean±SD. The gene expression of ECAD (*CDH1*), SLUG (*SNAI2*), and NCAM (*NCAM1*) was presented as median and interquartile range due to their asymmetrical distribution. Immunohistochemical expression was presented as frequencies and percentages. Noninvasive and invasive adenomas were compared using the chi-square and Mann-Whitney tests. The level of significance was set at 5% and data were analyzed using SPSS (USA), version 23.0.

## Results

Of 35 patients with acromegaly, 19 (54.3%) were women. Mean patient age was 47.1±13.4 SD years, ranging from 18 to 74 years. Immunohistochemically, 15 (42.9%) adenomas expressed GH alone, 14 (40%) expressed both GH and prolactin, and the remaining adenomas expressed GH and other hormones.

Regarding tumor grade based on preoperative images, 3 (8.6%) patients had grade I adenomas, 9 (25.7%) had grade II adenomas, 10 (28.6%) had grade III adenomas, and 13 (37.1%) had grade IV adenomas. Of these, 12 (34.3%) were noninvasive and 23 (65.7%) were invasive tumors. There was no statistically significant difference in gender or age between the noninvasive and invasive groups (P=0.135 and P=0.128, respectively).

### Immunohistochemical expression of ECAD and NCAM

Due to material availability, the samples of 30 patients were subjected to immunohistochemical analysis of protein expression. Of these, 11 (36.7%) were noninvasive tumors (3 grade I and 8 grade II tumors) and 19 (63.3%) were invasive tumors (8 grade III and 11 grade IV tumors).

**Figure 1.**
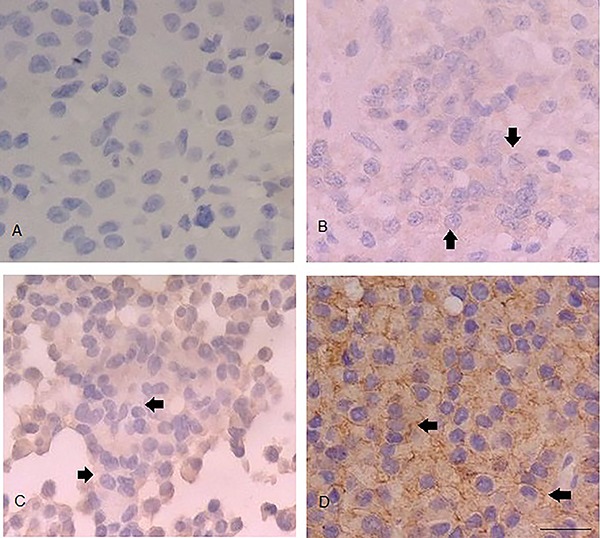
Immunohistochemical expression of ECAD (400×). *A*, Negative expression (−). *B*, Weak expression (+) (final score: 3). *C*, Moderate expression (++) (final score: 6). *D*, Strong expression (+++) (final score: 12). All represent invasive tumors. Arrows indicate immunopositivity in the plasma membrane. Bar=200 µm for all panels.

The samples of 29 (96.7%) patients expressed ECAD. Of these, 26 (86.7%) showed strong expression (+++), 2 (6.7%) showed moderate expression (++), 1 (3.3%) showed weak expression (+), and 1 (3.3%) showed no expression (Figure 1).

When analyzed according to tumor grade, ECAD was strongly expressed in 100% of grade I and II adenomas, 62.5% of grade III adenomas, and 90.9% of grade IV adenomas. Strong expression of ECAD was observed in 100% of noninvasive tumors and 78.9% of invasive tumors (Table 1).

**Table 1.** Immunohistochemical expression of ECAD and NCAM according to tumor grade and invasiveness based on preoperative images.

Grade	n=30	Immunohistochemistry							
		ECAD				NCAM			
		−	+	++	+++	−	+	++	+++
I	3	0	0	0	3	0	0	3	0
II	8	0	0	0	8	2	4	0	2
Total Noninvasive	11	0	0	0	11	2	4	3	2
III	8	1	1	1	5	1	4	3	0
IV	11	0	0	1	10	3	5	2	1
Total Invasive	19	1	1	2	15	4	9	5	1

Grade I, II, III, and IV (Asa and Ezzat) (13). Immunohistochemical expression: (−) negative, (+) weak, (++) moderate, (+++) strong.

**Figure 2.**
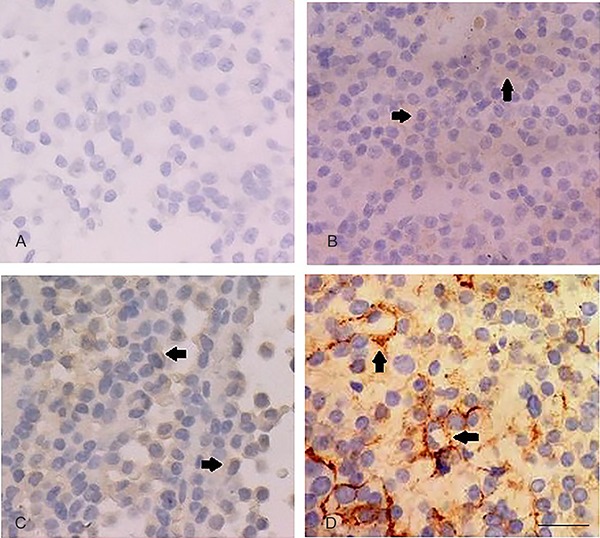
Immunohistochemical expression of NCAM (400×). *A*, Negative expression (−). *B*, Weak expression (+) (final score: 3). *C*, Moderate expression (++) (final score: 6). *D*, Strong expression (+++) (final score: 8). All represent invasive tumors. Arrows indicate immunopositivity in plasma membrane. Bar=200 µm for all panels.

The samples of 24 (80%) patients expressed NCAM. Of these, 3 (10%) showed strong expression (+++), 8 (26.7%) showed moderate expression (++), 13 (43.3%) showed weak expression (+), and 6 (20%) showed no expression (Figure 2).

When NCAM expression was analyzed according to tumor grade, grade I adenomas showed moderate expression in 100% of cases, grade II adenomas showed weak and strong expression in 75% of cases, grade III adenomas showed weak and moderate expression in 87.5% of cases, and grade IV adenomas showed weak, moderate and strong expression in 72.7% of cases (Table 1). Weak, moderate and strong expression of NCAM was observed in 81.8% of noninvasive tumors and 79% of invasive tumors (Table 1). There was no statistically significant relationship between tumor grade or invasiveness and immunohistochemical expression of ECAD (P=0.445) and NCAM (P=0.708). There was no statistically significant relationship between tumor grade or invasiveness and negative expression or any positive expression (weak, moderate, and strong) of ECAD (P=0.543) and NCAM (P=0.896).

### Gene expression of ECAD (CDH1), SLUG (SNAI2), and NCAM (NCAM1)

Due to RNA availability, the samples of 20 patients were analyzed for *CDH1* gene expression. Of these, 10 (50%) showed expression equal or higher compared to normal pituitary gland pool (median, 1.08). Of 12 samples analyzed for *SNAI2*, none showed gene expression. Of 15 samples analyzed for *NCAM1*, 8 showed gene expression (median, 1.14) (Table 2).

**Table 2.** Gene expression of ECAD (*CDH1*), SLUG (*SNAI2*) and NCAM (*NCAM1*).

	*CDH1*	*SNAI2*	*NCAM1*
Gene expression	(n=20)	(n=12)	(n=15)
n (%)	10 (50%)	0 (0%)	8 (53.3%)
Median	1.08	0.22	1.14
Interquartile range (25th–75th)	0.10–5.64	0.08–0.44	0.72–2.67
Noninvasive tumor	(n=9)	(n=6)	(n=7)
n (%)	5 (55.6%)	0 (0%)	3 (42.9%)
Median	1.20	0.27	0.84
Interquartile range (25th–75th)	0.83–5.20	0.09–0.52	0.33–2.19
Invasive tumor	(n=11)	(n=6)	(n=8)
n (%)	5 (45.5%)	0 (0%)	5 (62.5%)
Median	0.12	0.22	1.36
Interquartile range (25th–75th)	0.02–6.85	0.04–0.36	0.78–3.51

Data are reported in general samples and according to invasiveness based on preoperative images.

Among noninvasive tumors, 5 (55.6%) expressed *CDH1*, none expressed *SNAI2*, and 3 (42.9%) expressed *NCAM1*. Among invasive tumors, 5 (45.5%) expressed *CDH1*, none expressed *SNAI2*, and 5 (62.5%) expressed *NCAM1* (Table 2).

There was no statistically significant relationship between tumor grade or invasiveness and gene expression of *CDH1* (P=0.295), *SNAI2* (P=0.485), and *NCAM1* (P=0.463).

## Discussion

Epithelial-mesenchymal transition is an important process in embryonic development that has been associated with cancer progression and metastasis, and loss of ECAD is considered a key initial step in the transdifferentiation of epithelial cells into a mesenchymal phenotype (16–19). In the present study, 29 (96.7%) patients showed immunohistochemical expression of ECAD.

Several studies of pituitary adenomas have analyzed ECAD and reported positivity in most cases, albeit at varying levels. Yamada et al. (12) analyzed 40 nonfunctioning adenomas and found that 24 cases had moderate to strong staining. Kawamoto et al. (10) identified ECAD at different staining intensities in all pituitary adenomas investigated. Qian et al. (20), investigating functioning and nonfunctioning pituitary adenomas, detected immunohistochemical expression of ECAD in 70% of the sample. Fougner et al. (6) evaluated 80 patients with acromegaly and observed immunohistochemical expression of ECAD in 73% of cases. In the study conducted by Zhou et al. (7), analyzing the immunohistochemical expression of ECAD in 35 GH-secreting tumors, only 2 patients did not express ECAD.

In the present study, 50% of patients showed *CDH1* gene expression. Elston et al. (21) evaluated *CDH1* gene expression and found that, of 30 adenomas analyzed, only one did not show gene expression. Jia et al. (22) found a significant correlation between reduced *CDH1* gene expression and findings of tumor invasion and cystic lesions. In general, PCR methods have increased sensitivity and specificity compared to immunohistochemistry, and agreement between the two methods is poor. The lack of agreement is due to the fact that, despite being complementary techniques, each one analyzes different aspects of cell biology. The purpose of RT-PCR is to verify whether the gene that produces protein is active through mRNA analysis, whereas immunohistochemistry verifies the presence of protein. The morphology is preserved in the immunohistochemistry procedure, allowing for recognition of immunostaining heterogeneity and confirmation that the identified positivity is located in the tumor cells. On the other hand, the RT-PCR assay is a non-morphologic technique, and contamination of tumor mRNA with normal tissue, such as nontumoral pituitary cells and stromal cells may affect the results and cause discrepancies between the techniques. In the present study, higher positivity was observed in immunohistochemistry than in PCR. The significance of this finding may be related to the heterogeneity of tumor cells, the presence of mutation in a small proportion of tumor cells and disorders in post-transcriptional and post-translational physiological regulations, but the mechanisms involved in this process need further clarification. In addition, possibly, the amount of immunopositive cancer cells in the sample was too small to give positive results by RT-PCR when mRNA levels were compared to normal pituitary tissue. Furthermore, as a universal explanation, one can consider the heterogeneity of the tumor and the fact that the immunohistochemically analyzed tissue was not exactly the same tissue fragment examined by RT-PCR. Finally, the expression on mRNA and protein levels was not always synchronized (23,24).

Tumor invasion can be assessed by imaging techniques, such as MRI and CT scans or histologically. MRI is superior to CT scanning in defining the pituitary gland and the sellar region and its boundaries because of its multiplanar capability and its good soft tissue contrast. CT scan is also a useful tool and coronal scans provide the optimal view. CT may be indicated for patients with MRI restrictions, such as a pacemaker, intracranial metal clips, metal prostheses and phobia (1).

The role of ECAD in pituitary adenoma invasiveness is controversial. Reduced ECAD expression has been described in prolactinomas and is inversely associated with tumor size and invasion (21,25). Studies have demonstrated reduced immunohistochemical expression of ECAD in invasive and recurrent adenomas (4,6,7) as well as in invasive macroadenomas (20). In the study conducted by Yamada et al. (12), 40 nonfunctioning adenomas were analyzed and no significant difference was observed in the immunohistochemical expression of ECAD between tumors with and without cavernous sinus invasion. Likewise, other studies of pituitary adenomas have found no association between ECAD and tumor invasiveness (10,11,22), which is consistent with the findings of the present study.

SLUG is a member of the Snail family of transcription factors that acts to suppress ECAD expression and helps regulate tight and adherent junction stability, desmosome disassembly, and protease expression (8). SLUG overexpression leads to reduced ECAD expression, an important factor for epithelial-mesenchymal transition (26). *In vitro* and *in vivo* studies of different cancer cell lines have demonstrated that SLUG expression is correlated with loss of ECAD (*CDH1*) transcripts, which may influence the prognosis of patients (26–29).

There are few studies in the literature that report on SLUG expression in pituitary adenomas. In a study of 41 nonfunctioning adenomas, the expression of SLUG by immunohistochemistry, PCR and western blotting was significantly increased in invasive tumors (4). Jia et al. (22) investigated the presence of SLUG in 59 functioning and 36 nonfunctioning adenomas and identified an association of *SNAI2* gene expression with tumor invasiveness and bone destruction of the sella turcica. In the present study, no *SNAI2* expression was detected by the qRT-PCR method. Data interpretation is difficult because *SNAI2* expression is known to be regulated by microRNAs (a group of small non-coding RNAs) and the Wnt (wingless) signaling pathway, although the exact mechanisms of molecular regulation of *SNAI2* are unclear (30,31). Several genes are involved in the pathogenesis of pituitary adenomas, and changes in multiple genes are required for neoplastic transformation, which may reflect the heterogeneous behavior of these tumors (32). In addition to SLUG, other transcription factors, such as SNAIL, TWIST, ZEB1, and ZEB2, may inhibit ECAD transcription and contribute to epithelial-mesenchymal transition (33). Likewise, the methylation status of the *CDH1* gene is also responsible for gene silencing in certain pituitary tumors (32,34).

NCAM is a membrane glycoprotein that belongs to the immunoglobulin superfamily and plays a role in the proliferation, growth, differentiation and survival of different cell types (35). It is known that progression to neoplasia involves the ability to adhere and interact with the surrounding cells and extracellular matrix, and loss or adhesion difficulties may be a determinant of epithelial neoplasia (36). Studies have demonstrated immunohistochemical expression of NCAM in pituitary adenomas, including GH-secreting tumors (5,37–39).

Regarding invasiveness, Kleinschmidt-DeMasters et al. (38) analyzed 20 pituitary adenomas and found no correlation between NCAM expression and tumor invasiveness, suggesting that this protein is not a useful marker of tumor invasiveness. In the study conducted by Trouillas et al. (5), only polysialylated NCAM (PSA-NCAM) was expressed in 46.3% of adenomas and in 85% of invasive tumors. Similar to our results of 53% of the sample with *NCAM1* gene expression, Rubinek et al. (40) showed, by means of RT-PCR, that this gene was expressed in 67% of GH-secreting adenomas, in only a minority of prolactin-secreting tumors, and in normal pituitary tissue.

In conclusion, the characterization of GH-secreting pituitary adenomas allows the early identification of patients with tumors with a high risk of recurrence or resistant to conventional therapy, thus providing a basis for the development of patient-tailored treatment strategies and follow-up. The absence of *SNAI2* overexpression and of *CDH1* and *NCAM1* suppression suggests that they are not associated with tumor invasiveness in GH-secreting pituitary adenomas. Further studies with a larger sample size are needed to better demonstrate the interrelationship of ECAD, SLUG, and NCAM and to determine the influence of these and other markers on the pathogenesis of acromegaly and tumor invasiveness.
